# Identifying the mechanisms of intron gain: progress and trends

**DOI:** 10.1186/1745-6150-7-29

**Published:** 2012-09-10

**Authors:** Paul Yenerall, Leming Zhou

**Affiliations:** 1 Department of Biological Sciences, University of Pittsburgh, Pittsburgh, PA, 15260, USA; 2 Department of Health Information Management, University of Pittsburgh, Pittsburgh, PA, 15260, USA; 3 Department of Bioengineering, University of Pittsburgh, Pittsburgh, PA, 15260, USA

**Keywords:** Intron, Intron gain, Intron evolution, Gene structure, Evolution, Mechanism

## Abstract

**Abstract:**

Continued improvements in Next-Generation DNA/RNA sequencing coupled with advances in gene annotation have provided researchers access to a plethora of annotated genomes. Subsequent analyses of orthologous gene structures have identified numerous intron gain and loss events that have occurred both recently and in the very distant past. This research has afforded exceptional insight into the temporal and lineage-specific rates of intron gain and loss among various species throughout evolution. Numerous studies have also attempted to identify the molecular mechanisms of intron gain and loss. However, even after considerable effort, very little is known about these processes. In particular, the mechanism(s) of intron gain have proven exceptionally enigmatic and remain topics of considerable debate. Currently, there exists no definitive consensus as to what mechanism(s) may generate introns. Because many introns are known to affect gene expression, it is necessary to understand the molecular process(es) by which introns may be gained. Here we review the seven most commonly purported mechanisms of intron gain and, when possible, summarize molecular evidence for or against the occurrence of each of these mechanisms. Furthermore, we catalogue indirect evidence that supports the occurrence of each mechanism. Finally, because these proposed mechanisms fail to explain the mechanistic origin of many recently gained introns, we also look at trends that may aid researchers in identifying other potential mechanism(s) of intron gain.

**Reviewers:**

This article was reviewed by Eugene Koonin, Scott Roy (nominated by W. Ford Doolittle), and John Logsdon.

## Background

Spliceosomal introns are segments of RNA that are excised by the spliceosome during the processing of pre-mRNA in eukaryotes. Although spliceosomal intron density varies widely among eukaryotes, no true eukaryote has ever been identified without a spliceosomal intron and some remnant of the spliceosome. Conversely, units of the spliceosome and/or spliceosomal introns have never been identified in any prokaryote [1,2]. Spliceosomal introns (herein referred to simply as introns) were originally believed to be “junk” DNA as they were not translated. However, since their initial discovery, numerous functional roles for introns have been elucidated, such as augmenting proteome diversity by enabling alternative splicing [3], enhancing gene expression [4-6] and harboring various *cis* and *trans* regulatory elements [7,8].

As researchers began to compare the structure of orthologous genes, it quickly became apparent that introns may be gained or lost throughout evolution [9]. Subsequent analyses have identified thousands of intron gains and losses [10-29]. These findings have sparked considerable interest into identifying the molecular mechanisms of intron gain and loss [11-15,18,20-24,27,30-35]. Two definitive mechanisms of intron loss, ***R****everse****T****ranscriptase-****M****ediated****I****ntron****L****oss* (RTMIL) and genomic deletions, have been identified and are widely accepted [36,37]. The definitive mechanism(s) of intron gain, however, remain elusive and controversial. All together, there have been at least seven commonly purported mechanisms of intron gain: *Intron Transposition*[38], *Transposon Insertion*[39], *Tandem Genomic Duplication*[40], *Intron Transfer*[31], *Intron Gain during Double-Strand Break Repair (DSBR)*[27], *Insertion of a Group II Intron*[38] and *Intronization*[41,42]*.*

Although seven possible mechanisms of intron gain have been proposed, researchers have identified thousands of novel introns whose mechanistic origins defy these explanations [11,17,18,22,23,27,28,43]. Surprisingly, even many recently gained introns, which have the highest probability of revealing their mechanistic origin, do not appear to have arisen via any of these mechanisms [27,44]. These findings raise an intriguing question: do these proposed mechanisms of intron gain fail to describe the mechanistic origin of so many novel introns because they are not genuine mechanisms of intron gain, or are there other process(es) generating novel introns? Here we review the proposed mechanisms of intron gain and summarize any previously identified direct (molecular) and/or indirect (intron gains identified during genomic analyses with purportedly known mechanistic origins) evidence that supports or refutes the occurrence of each of these proposed mechanisms. Furthermore, we examine trends that may aid researchers in identifying other novel mechanism(s) of intron gain.

## Review

### The proposed mechanisms of intron gain

#### Intron transposition

**Figure 1:**
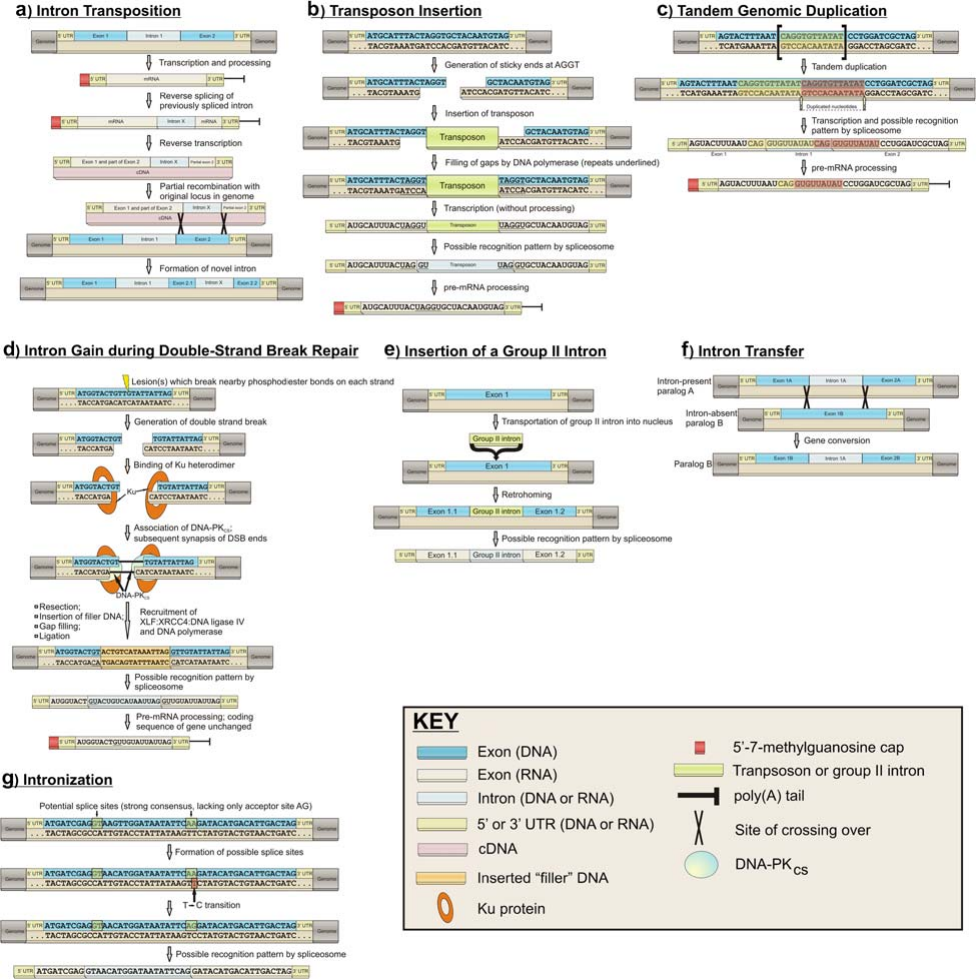
**The seven proposed mechanisms of intron gain and loss.** Introns shown are shorter in length than necessary for splicing strictly for illustrative purposes. **a**) Intron Transposition with “partial” recombination. Other routes of intron transposition (discussed in text) by which concurrent intron gains/losses may occur can be also envisioned. **b**) Transposon Insertion. Imprecise intron gain may also be envisioned (discussed in text). **c**) Tandem Genomic Duplication using duplicated AGGT sequences for splice sites. The segment to be duplicated is flanked by brackets. The template for the duplicated nucleotides is highlighted in yellow; the duplicated nucleotides are highlighted in red and underlined. Imprecise intron gain may also frequently occur (discussed in text). **d**) Intron Gain during Double-Strand Break Repair. Protein names are from mammals. Short direct repeats created by gap filling are underlined. The possible pathways of non-homologous end joining (NHEJ) are from proposals in [45-48]. **e**) Insertion of a Group II Intron. **f**) Intron Transfer. **g**) Intronization. Strong consensus donor and acceptor sites (following a single point mutation) are highlighted in yellow. The place at which the hypothetical point mutation occurs is highlighted in red.

The most commonly purported mechanism of intron gain is intron transposition [34]. Intron transposition has been proposed to occur when a spliced intron reverse splices into either its own mRNA or another mRNA at a previously intron-less position. This intron-containing mRNA is then reverse transcribed and the resulting intron-containing cDNA may then cause intron gain via three different routes: it may undergo “complete” or nearly complete recombination with its original genomic locus, in which recombination between the intron-containing cDNA and the original genomic locus occurs both in exonic and intronic regions (if the gene contains introns), thereby deleting any introns within the region of recombination and causing concurrent intron gain and loss; it may undergo “partial" recombination, in which recombination only occurs between the intron-containing cDNA and an exonic region, avoiding the deletion of introns; or it may retropose into the genome at a different locus and then transfer the gained intron via “partial” or “complete” recombination to the original locus. Partial recombination between the intron-containing cDNA and the original genomic locus is the most commonly discussed route of intron transposition and is illustrated in Figure 1a.

Intron transposition has frequently been favored among the seven proposed mechanisms of intron gain by researchers [34,37], likely because a transposed intron would already harbor the donor, acceptor, and branch point splicing sequences necessary for splicing [49]. However, the molecular process(es) by which intron transposition may occur, if it occurs at all, are unknown. To an extent, the most crucial and nebulous step in this process, reverse splicing, has been shown to occur [50]. However, this has only been demonstrated under non-physiological conditions using a mutant of Prp22 that fails to release the mRNA from the spliceosome. Furthermore, because a mutant was used that failed to release the mRNA, this process has only been shown to reverse splice introns back into their original position [50]. The process by which a previously spliced intron and spliceosome may bind (or remain bound), recognize an mRNA (based upon sequence motifs, protein complexes loaded onto the mRNA or random interactions), and begin nucleophilic attack and subsequent reverse splicing, is not known. Given that a recent study identified 95 introns with homologous sequences, of which only 11-24% may be novel introns [44], it is possible that interactions between proteins recruited to and deposited on the mRNA during and after splicing [51-53] may recruit spliceosomes “loaded” with previously spliced introns. Thus, reverse splicing may occur frequently, if not entirely, at positions in the mRNA that have already undergone splicing (i.e. only pre-existing intronic positions). This potential preference, if true, may help explain the finding of supposed parallel intron gains [27] if, following an initial intron gain, a different intron is transposed into this intronic position and both intronic sequences are maintained in the species. Alternatively, it is possible that reverse splicing occurs randomly at any position in an mRNA; however, the finding that the majority of suspected reverse splicing events occurred at previously intronic positions [44] argues against this explanation.

Even if intron transposition does occur, it has been suggested that any mechanism of intron gain that relies upon reverse transcriptase (RT) may not be a prevalent mechanism of intron gain. One potential problem is known as the “rate paradox” [34]. This may occur because the presumed most prevalent mechanism of intron loss, RTMIL [35], occurs via a process nearly identical to intron transposition, but does not require reverse splicing. Thus, the difference between the genome-wide rate of intron gain via intron transposition and intron loss via RTMIL should equal the rate of reverse splicing. However, as pointed out by Roy and Irimia, reverse splicing is believed to be an extremely rare process, as no reverse spliced introns have been found in any EST or cDNA sequences [34]. Furthermore, a recent statistical analysis has shown that it is unlikely that RT played a prominent role in intron gain throughout evolution [35].

Other proposed molecular mechanisms of intron gain that mimic intron transposition, such as spliceosomal retrohoming or reverse transcription template switching [34], may occur and may be responsible for a number of intron gains that have been identified with sequences homologous to other introns [25,44]. However, both of these models rely upon RT and therefore suffer from some of the same problems as the canonical route of intron transposition, as discussed above. Regardless of the exact molecular process, indirect evidence exists which suggests that some form of intron transposition is likely a genuine mechanism of intron gain (Table 1).

**Table 1:** Intron gains identified with a purportedly known mechanistic origin

**Proposed mechanism**	***In vivo*demonstration**	**Indirect evidence**	**Number of events unambiguously identified**	**Specie(s) event identified in**
Intron Transposition	No	Yes [25,44]	14	*Mycosphaerella*, *Oikopleura*
Transposon Insertion	No	Yes [23,25,54,55]	35	*Oryza*, *Drosophila, Oikopleura, Zea*
Tandem Genomic Duplication	Yes [56]	Yes [18,57]	188	*Arabidopsis, Oryza, Caenorhabditis, Drosophila,* mouse, human, *Cryptococcus*
Intron Gain during DSBR	No	Yes [22,23,27,58,59]	5	*Drosophila, Daphnia, Aspergillus, Bigelowiella,* human
Intron Transfer	No	Yes [22,31,44]	3	*Mycosphaerella, Aspergillus, Chironomus,*
Insertion of a Group II Intron	Likely does not occur [60]	No	0	
Intronization	No	Yes [41,42,61,62]	29	*Cryptococcus*, *Caenorhabditis*

Only intron gains with novel splice sites (i.e. not simple duplications of pre-existing introns) were included. Only novel introns with EST support were selected from [57]. For a more detailed treatment of events, see the MIGL database located at http://cpath.him.pitt.edu/intron/index.php (manuscript in preparation).

#### Transposon insertion

Transposon insertions into genic regions are generally viewed as deleterious mutations. However, one underappreciated outcome of this process may be intron creation. Such an insertion may completely intronize the transposon without disrupting the coding sequence when a transposon inserts into the sequence AGGT, resulting in the duplication of this sequence on each side of the transposon (illustrated in Figure 1b). Three lines of evidence support the generation of introns via this model. One is that the sequence which the transposon inserts into, AGGT (also known as a protosplice site [63]), is believed to be a site of preferential intron gain [63-66]. Another is that the donor and acceptor splice sites created by this insertion, specifically the donor site AG|GT and the acceptor site AG|G (where “|” specifies splice junctions), adhere to the consensus donor and acceptor splice sites found in many organisms [67-71]. Finally, if these potential donor and acceptor splice sites are utilized efficiently by the spliceosome, any transposon may insert into the sequence AGGT in any gene without altering the genes coding sequence (demonstrated in Figure 1b). Alternatively, the transposon itself may harbor strong donor and acceptor splice sites near its boundaries or activate nearby latent splice sites, enabling its precise, or nearly precise, excision by the spliceosome. Indirect evidence of intron gain via transposon insertion has existed for nearly 20 years (see Table 1). However, what is not understood is exactly why these elements are spliced (if not by pure chance), or if any transposons preferentially cause intron gain, potentially due to target site preferences and/or the sequence of the transposon itself.

#### Tandem genomic duplication

Although widely underappreciated, the tandem genomic duplication of an exonic segment has recently emerged as a genuine and potentially prevalent mechanism of intron gain. Due to the similarity between consensus donor and acceptor splice sites, both of which closely resemble the sequence AGGT, the tandem genomic duplication of an exonic segment harboring the sequence AGGT generates two strong potential splice sites. If these splice sites are utilized by the spliceosome, the sequence between the original and duplicated AGGT will be spliced, affording the “precise” generation of an intron, i.e. the creation of an intron without alteration of the coding sequence of the gene (illustrated in Figure 1c). As discussed in the Transposon Insertion section above, use of AGGT as both the donor and acceptor splice site conforms to the most common splice site consensus sequences found in a variety of organisms [67-71], and AGGT has been found to be a site of preferential intron gain [64,65]. In contrast to precise intron gain, in which the coding sequence of the gene remains unaltered, tandem genomic duplication may also result in “imprecise” intron gain, in which the coding sequence is altered. This may occur when latent splice sites within the duplicated region are utilized, resulting in the addition of nucleotides to the coding sequence of the gene. Alternatively, the duplication may result in the activation of latent splice sites near the duplicated sequence, resulting in the removal of nucleotides from the coding sequence.

Unlike any other proposed mechanism of intron gain, *in vivo* evidence exists to support the occurrence of this mechanism. The ability of this mechanism to have produced a novel intron nearly 500 million years ago in the ancestor of jawed vertebrates was recently tested and verified *in vivo*[56]. Not only has this process been shown to be a feasible mechanism of intron creation *in vivo*, but a plethora of indirect evidence in support of this mechanism has also been identified in a number of eukaryotes (Table 1). It should be noted, however, that many of these intron gains were imprecise and resulted from the activation of latent splice sites within the duplicated segment [57].

#### Intron Gain during Double-Strand Break Repair (DSBR)

Double-strand breaks (DSBs) are genomic lesions in which nearby phosphodiester bonds are severed on both strands of the DNA double helix. Such lesions may be caused by ionizing radiation, reactive oxygen species, or cellular processes. Because the repair of these lesions is paramount to cell viability, organisms have evolved two ubiquitous, genetically distinct, well conserved processes to repair these breaks: homologous recombination and non-homologous end joining (NHEJ) [72]. The former results in the precise repair of the break and requires an undamaged template, such as a sister chromatid. Thus, homologous recombination occurs primarily during the S and G2 portions of the cell cycle [73]. NHEJ, on the other hand, requires no template. If the DNA ends are undamaged and complementary, NHEJ faithfully restores the break point junction. However, if the DNA ends are damaged or are not complementary, NHEJ may insert or delete nucleotides from the break point junction [45,46]. DSBR by NHEJ was recently implicated in intron gain when researchers identified short direct repeats flanking 43% of gained introns in *Daphnia*[27]. These repeats suggest that these introns were gained by the insertion of nucleotides during the repair of staggered DSBs by NHEJ [27] (illustrated in Figure 1d). Because NHEJ has been shown to preferentially insert mitochondrial DNA [74,75], further support for this model was garnered when the authors identified a gained intron that was homologous to the mitochondrial 16S ribosomal subunit [27]. How or why NHEJ preferentially uses mitochondrial DNA [74,75], why these sequences appear to integrate preferentially into genic regions [58], and why these sequences may function as introns, if not purely by chance, are topics that await further investigation.

Currently, indirect evidence for this proposed mechanism consists of gained introns with high similarity to mitochondrial DNA, rather than nuclear DNA (Table 1). In an attempt to determine the frequency by which introns without similarity to mitochondrial DNA may be gained via NHEJ, researchers have also quantified the number of repeats found flanking gained introns. In comparison to conserved introns, some studies have found gained introns to be enriched for repeats near their splice junctions [22], while others have not [23,44]. The current incongruence of gained introns to be preferentially flanked by repeats may be the result of a number of factors, such as differences in NHEJ among species [30,76-78] and cell types [79], insufficient sample sizes, or repeats and/or introns being inserted via other mechanisms. Alternatively, sequences of DNA inserted by NHEJ that are long enough to potentially form introns [80] may not frequently be flanked by direct repeats (see, for instance, inserts >30 bp long in [58,75,81,82], however, also see one insert in [83]). Finally, it should be noted that other studies have identified a number of gained introns that are flanked by repeats [27,28]. However, these numbers must be compared to the number of conserved introns flanked by repeats. This ensures that the number of repeats found flanking gained introns is significantly higher than the background level of repeats found to naturally flank introns. For instance, in Drosophila we found that 25% of gained introns were flanked by direct repeats ≥ 5 bp; however, 26% of conserved introns were flanked by repeats of the same size [23], indicating that while many introns are flanked by repeats in Drosophila, there is no bias for gained introns to be flanked by repeats in Drosophila.

#### Insertion of a Group II intron

Group II introns are self-splicing introns found in bacterial genomes and the organellular genomes of many eukaryotes. A number of remarkable similarities exist between group II introns and spliceosomal introns, such as their method of excision from a primary transcript, the sequence of their 5’ and 3’ ends, and structural similarities [84]. Even as our understanding of introns lay in its infancy, these similarities gave rise to the idea that spliceosomal introns and group II introns may be evolutionarily related [85]. Subsequent investigations have shown that it is likely that, following endosymbiosis between an α-proteobacteria and its archaeal host, many group II introns were transferred from the genome of the α-proteobacteria to the archaeal genome. The invasion and subsequent degradation of these elements may have imposed selective pressures which eventually gave rise to various hallmarks of eukaryotic cells, such as the nucleus [86,87], nonsense-mediated decay [60,88], and spliceosomal introns themselves [1,89,90].

In light of the findings that group II introns were likely the progenitors of spliceosomal introns, the retrohoming, or insertion, of a group II intron into a nuclear gene was proposed to cause recent spliceosomal intron gain [38] (Figure 1e). While it is widely believed that group II introns originally gave rise to spliceosomal introns, a recent *in vivo* assay demonstrated that the insertion of a group II intron into a nuclear gene nearly abolishes gene expression [60]. Thus, the evolution of nucleus-cytosol compartmentalization and nonsense-mediated decay following the initial invasion of group II introns (and subsequent creation of spliceosomal introns) may now impede intron gain via this mechanism. In accordance with these findings, indirect evidence of a recent intron gain via the insertion of group II intron has never been identified (Table 1). Therefore, it is unlikely that the insertion of a group II intron into a nuclear gene is a mechanism of recent spliceosomal intron gain. It is interesting to note, however, that the insertion of a group I intron into a nuclear gene does not appear to affect gene expression [60].

#### Intron transfer

Intron transfer has been hypothesized to result in intron gain when a paralog or pseudogene gains an intron and then transfers this intron via recombination to an intron-absent location in its sister paralog (illustrated in Figure 1f) [31]. Although indirect evidence has been found to support intron transfer (Table 1), this mechanism does not explain how the initial intron was gained, but rather explains how once gained an intron may propagate to other paralogs. It is possible that initially an imprecise intron gain occurs in a paralog or pseudogene and, following suppressor mutation(s), this intron is transferred via recombination to another paralog. Such a mechanism may allow introns to be initially gained imprecisely in a duplicated, unessential copy of a gene. Then, following suppressor mutation(s) in this neutrally evolving, extra intron-containing duplicate gene, recombination between the intron-containing region in the duplicated gene and the functional gene may result in precise or near precise intron gain in the essential copy of the gene. This process would allow an initially deleterious intron gain event in one paralog to result in non-deleterious intron gain in both paralogs without imposing a negative fitness cost to the host. If true, this suggests that intron gain rates should be higher in paralogous genes than genes without paralogs. This prediction has been confirmed in a broad range of species [15,91,92].

#### Intronization

Intronization is the process by which mutations create novel introns from formerly exonic sequence. Thus, unlike other proposed mechanisms of intron gain, this mechanism does not require the insertion or generation of DNA to create a novel intron. In the most commonly discussed route of intronization, mutations, generally point mutations, forge novel splice sites from exonic sequence [41,42], resulting in the formation of a novel intron (Figure 1g). An alternative route of intronization has been proposed to occur when, in an exonic segment flanked by latent splice sites, a premature termination codon is generated via mutations [93]. The spliceosome may then act upon these latent splice sites in a mysterious process known as nonsense-associated altered splicing [94-97], removing the premature termination codon from the transcript and saving it from destruction via nonsense-mediated decay. This differentially spliced product (i.e. lacking the premature termination codon) has then been proposed to persist until subsequent mutations facilitate efficient utilization of these latent splice sites by the spliceosome, resulting in the formation of a novel intron [93].

Only the most commonly discussed route of intronization, by which mutations forge novel splice sites, has garnered indirect evidence (Table 1). This may be because the alternative route of intronization is much more difficult to detect. Alternatively, this route may not occur. Regardless, nonsense-mediated decay may play some role in enabling intron gain, potentially by facilitating the persistence of an initially infrequently spliced gained intron that harbors a premature termination codon, as a recent study in Drosophila found that novel introns were enriched for in-frame stop codons [28], although it should be noted that similar results were not found in *Aspergillus*[22]. Furthermore, it is possible that the point mutations that generate potential splice sites during intronization may, at least initially, be utilized infrequently. Thus, the ability to detect intronization events relies heavily upon accurate gene annotation. Additionally, as these point mutations may not initially generate strong splicing signals, alternative splicing may occur using these or other latent splice sites, resulting in transient bouts of intronization. Such a process may confound both the process of genome annotation and analyses aimed at identifying intron gains and losses. Therefore, identification of many of these events has been limited to species with deep transcriptome profiling [41,42] or in analyses of recently created genes [61,62]. Indirect evidence of intronization may exist in fungi [22] as well as other species, but has yet to be identified. Further analyses armed with extensive RNA-seq data may shed further light on the prevalence of intronization in other species.

### Trends in intron gain

Given that thousands of gained introns have been identified with no known mechanistic origin, it is apparent that the seven proposed mechanisms of intron gain fail to describe how the vast majority of novel introns have arisen. Because many gained introns have sequences that are not homologous to any endogenous sequences, it is likely that other mechanism(s) are also causing intron gain. Therefore, we must look at trends in intron gain that may lead researchers towards a different mechanistic explanation.

Perhaps the most tantalizing trend in intron gain is the role that transcription may play in intron gain. Studies in organisms with a dedicated germline have shown that genes that have experienced intron gain events are enriched for germline expression [23,24]. Other studies have shown that intron gain rates positively correlate with expression levels [25,98]. Furthermore, a recent study identified thousands of introns that appear to have been created by repeats, dubbed introner elements, in the *Micromonas* isolate CCMP1545 [99]. These repeats lack transposable element characteristics and were only found co-linear to transcribed DNA, suggesting that these repeats were conceived via a transcription-based mechanism. Much like the introner elements identified in *Micromonas*, another recent study in fungi identified elements that create introns, dubbed introner-like elements. These elements were also only found co-linear to transcribed DNA and also did not appear to be canonical transposable elements [100]. However, these elements were shown to have originated from a singular element, to be absent from species that have undergone intron transposition [25] and, while they were spliced efficiently, they were shown to rapidly degenerate into normal spliceosomal introns, indistinguishable from their original source [100]. Thus, throughout evolution, introner-like elements may have arisen in various species, caused brief episodes of massive intron gain, become silenced by an as of yet uncharacterized mechanism, and then rapidly degenerate, leaving behind no trace of the mechanism by which these introns arose [101]. If true, this would explain the varied and occasionally punctuated rates of intron gain found in some species [1,29]. Most importantly, unlike introner elements, introner-like elements are predicted to fold into stable RNA secondary structures, suggesting that these elements propagate via an RNA intermediate [100]. Taken together, these results suggest that either the act of transcription and/or the transcript itself may play an important and as of yet undefined role in intron gain. Given that RT does not appear to have played a major role in intron gain throughout evolution [34,35], and that the newly identified introner-like elements appear to propagate via an RNA intermediate and are only found in transcribed regions [100], it is more likely that the act of transcription and/or the transcript itself, without being converted to cDNA, enables intron gain. The exact process(es) by which transcription may facilitate or cause intron gain, if it occurs at all, warrants further investigation.

Frequently a positional bias has been used to support possible mechanisms of intron gain or loss. A bias for intron gains or losses in the 3’ end of genes suggests that RT may have played a role in these events because RT transcribes from 3’ to 5’ and is known to frequently dissociate from templates before reaching their 5’ ends [102,103]. Overall, there is discordance among species as to the positional bias for intron gain: in some species intron gains appear biased towards the 5’ end of genes [17,23,28]; in others intron gains appear biased towards the 3’ end of genes [10]; and others appear to have no detectable bias [13,25,35,44]. This discrepancy suggests that intron gain may not rely heavily upon any mechanism that suffers from a positional bias. Alternatively, mechanisms of intron gain that favor 5’ or 3’ gain may operate in some species but not in others.

## Conclusion

Out of seven proposed mechanisms of intron gain, six have indirect evidence to support their occurrence (Table 1). The only proposed mechanism of recent intron gain that lacks any indirect evidence is the insertion of a group II intron. The insertion of a group II intron into a nuclear gene has also been shown to nearly abolish gene expression *in vivo*[60], suggesting that while group II introns were likely the progenitors of modern spliceosomal introns, they no longer create spliceosomal introns. Only one proposed mechanism of intron gain, tandem genomic duplication, has been shown to have been a genuine mechanism of intron gain *in vivo*[56]. Furthermore, this mechanism has a plethora of indirect evidence (Table 1), strongly suggesting that this mechanism is a prevalent and ubiquitous mechanism of intron gain in many species. The testing of other proposed mechanisms *in vivo*, in particular intron gain during DSBR, intron transfer and intronization, is feasible. Demonstration of these mechanisms *in vivo* is essential to solidify them as genuine mechanisms of intron gain. Further genomic analyses, especially those performed at the population level, may then quantify the relative contribution of each mechanism, potentially identifying species-specific biases that may help account for the varied rates of intron gain among species [1,29].

Significant progress has been made in identifying the mechanisms of intron gain. However, this field still lies in its infancy. Even with one definitive and five likely mechanisms of intron gain, the vast majority of gained introns lack a known mechanistic origin. Therefore, it is essential that other mechanisms are envisioned and tested, as it is likely that undiscovered mechanism(s) of intron gain exist. Germline expressed and highly expressed genes tend to accumulate introns [23-25,98]; yet, RT does not appear to have played a major role in intron gain [35]. Furthermore, a recent study identified intron creation via introner-like elements, which have been posited to have potentially created introns in species other than fungi and propagate via an RNA intermediate [100]. Hence, it is possible that an undiscovered mechanism of intron gain exists that relies upon either the act of transcription and/or the transcript itself. It is also possible that some other completely uncharacterized molecular mechanism is responsible for recent intron gains, or that novel introns are being obtained from unknown exogenous sources, such as viruses, bacteria or mobile genetic elements that have not yet been sequenced or identified. A combination of *in vivo* assays and genomic analyses performed at the population level, which will likely identify intron gains before extensive sequence divergence obscures their mechanistic origin, will likely prove the most fruitful avenues towards identifying and understanding the molecular processes underlying intron gain.

## Abbreviations

DSBR: Double-strand break repair; NHEJ: Non-homologous end joining; RT: Reverse transcriptase; RTMIL: Reverse Transcriptase-Mediated Intron Loss; DSB: Double-strand break.

## Competing interests

The authors declare that they have no competing interests.

## Authors’ contributions

PY drafted, illustrated and revised the manuscript. LZ revised the manuscript. All authors read and approved of the final version of the manuscript.

## **Reviewers’ comments**

Reviewer #1: Dr. Scott Roy (nominated by Dr. W. Ford Doolittle)

*Reviewer’s comments*: The authors provide a very useful and timely review on the mechanisms of intron gain, a field in which a tremendous amount of progress has been made in the past few years. I have very few criticisms, and even on those points where I might have a difference of opinion in regards to emphasis or interpretation, I think the authors' perspective is well supported and balanced, and so overall I do not think any changes need to be made.

*Authors’ response*: We thank you for your review and comments. Because most of these mechanisms lack definitive evidence to support how/if they occur, we agree that there exist various, and at times conflicting, opinions and interpretations upon how/if these proposed mechanisms occur. Thus, we are delighted that you feel our presentation was balanced and supported by primary literature.

Reviewer #2: Dr. Eugene Koonin

*Reviewer’s comments*: This is a timely review of the important and fascinating problem of intron gain routes and mechanisms. The article is very well structured around the 7 distinct (proposed) routes of intron gain. My mild disappointments have to do with the excessive brevity of some of the sections. In particular, the section on Group II intron insertion is succinct to the point of being potentially misleading. I agree with the authors that there is no indication of recent intron gain via Group II intron insertion. However, this does not put into doubt the ultimate origin of spliceosomal introns from Group II introns whereas from the current version, the impression is that the authors refute that scenario. The situation can be easily remedied with a brief recapitulation of the evidence in support of the evolutionary links between self-splicing and spliceosomal introns.

*Authors’ response:* We intentionally kept our review brief in order to meet the word limit of reviews in this journal (~3000 words). We apologize if, in our desire to write succinctly, we may have accidently expressed our beliefs and prior findings incorrectly, especially in regards to the origin of spliceosomal introns. As you mentioned, a significant amount of evidence exists to support the theory that group II introns, originating from an α-proteobacterium that would later go onto serve as the mitochondria, invaded the genome of their archaeal host, and in response to this invasion, selective pressures eventually gave rise to the nucleus, nonsense-mediated decay, and spliceosomal introns. We do note that in our original manuscript we directly stated that “group II introns are widely regarded as the progenitors of spliceosomal introns”. However, the wording of this section may have been misleading. Thus, we have rewritten the section “Insertion of Group II Intron” to better exemplify this point and have also added a brief primer on the origin of spliceosomal introns.

*Reviewer’s comments*: In addition, the authors do not explicitly address the connection between alternative splicing and intron gain mechanisms. Emergence of a new alternative splice form often involves ("part-time") intronization, so I think this belongs in the paper.

*Authors’ response*: Alternative splicing and, in particular, nonsense-mediated decay likely play key roles in the evolution of gained introns by alleviating negative fitness costs imposed upon organisms following the initial intron gain event (or, more explicitly stated, the initial mutation). However, due to space limitations, we chose to focus only on the mechanisms that may initially cause the intron gain (the initial mutation). The forces at play following this mutation that fixate this mutation in a population/species deserve a more in-depth treatment than we could provide with the space allotted. Regardless, we have added a brief discussion of these topics to the “Intronization” section, as this mechanism may rely most heavily upon evolutionary processes to yield efficient introns.

*Reviewer’s comments*: Again, a very timely and useful review but with a little more attention to details, it could become even better.

*Authors’ response*: We thank you for your review and apologize if we may have neglected some topics in an effort to be succinct.

Reviewer #3: Dr. John Logsdon, University of Iowa

This reviewer provided no comments for publication.
